# Audience responses to and interactional dynamics of transnational digital narratives by international students in China: a thematic and configurational analysis of YouTube comments

**DOI:** 10.3389/fpsyg.2026.1976786

**Published:** 2026-10-19

**Authors:** Ning Wang, Jinling Wang

**Affiliations:** 1 Department of Design, Kyungpook National University, Daegu, Republic of Korea; 2 School of Languages and Media, Anhui University of Finance and Economics, Bengbu, China

**Keywords:** audience responses, BERTopic, fsQCA, interactional recontextualization, international students in China, transnational experiential narratives

## Abstract

Digital platforms are reshaping the configuration of communicative actors and the processes through which meaning is produced in transnational communication. Individuals with sustained cross-cultural living experience have also become important communicative actors connecting different social experiences with public understanding. Existing research has paid considerable attention to the digital expressions of transnational individuals and audience evaluations of such content. However, further investigation is needed into how transnational experiential narratives are understood by audiences after entering platforms, how the focus of responses changes in subsequent interaction, and how different response structures are associated with platform communication performance. Drawing on YouTube videos produced by international students in China and their comment data, this study employs BERTopic-based topic identification, analysis of thematic correspondence between comments and replies, and fuzzy-set qualitative comparative analysis (fsQCA) to examine audience response structures, interactional changes, and their relationships with platform communication performance. The findings reveal that transnational experiential narratives generate a multilayered structure of audience responses within the comment space, ranging from relational interaction and the practical use of lived experience to risk appraisal and national and societal interpretation. Between comments and associated replies, there is both thematic retention and a substantial proportion of thematic transition. Concrete experiences of living and studying more often serve as starting points for subsequent discussion, while responses become more concentrated around relational-affective engagement and educational information seeking at the reply stage. Configurational analysis further shows that higher composite communication performance does not correspond to any single response theme but is associated with specific combinations of multiple audience responses. In particular, the lower salience of concrete study-abroad-life discussion and the higher salience of national and transnational interpretive frames are relatively stable features across different minimization solutions. The study shows that the communicative meaning of transnational experiential narratives is not fixed once creators complete their expression, but is continuously generated through audience understanding, response, and interaction. Their communication performance is therefore better understood through the combinational relationships among multiple audience responses than through single-theme effects.

## 1 Introduction

Social media platforms and their algorithmic distribution mechanisms are reshaping the production, distribution, and participatory structures of international communication. In a platformized communication environment, the transnational circulation of information no longer depends primarily on content produced and disseminated unidirectionally by professional institutions. Instead, its communicative visibility and public influence are increasingly shaped by the combined effects of platform mechanisms and user participation (Nieborg and Poell, 2018; van Dijck et al., 2018). At the same time, social media have lowered the barriers to participation in transnational content production and dissemination, enabling ordinary creators, transnationally mobile individuals, and other non-state actors to participate more extensively in international information flows (Ingenhoff et al., 2021; Li and Feng, 2022). The expansion of communicative actors and content supply, however, does not mean that the meanings originally intended by creators will reach audiences in a stable and unchanged form. After content enters a platform, it is further interpreted and assigned meaning through audience understanding, evaluation, and interaction, shifting meaning production from one-way transmission toward participatory interpretation and negotiation (Lomborg, 2015; Picone et al., 2019). Research on international communication must therefore consider not only who communicates and what content is produced, but also how that content is understood and endowed with meaning after entering audience spaces.

As the actors engaged in transnational communication have become increasingly diverse, individual creators have drawn on their own cross-cultural experiences and social encounters to participate in the representation and interpretation of foreign societies. Their experiential positions have consequently become an important basis shaping both narrative content and audience understanding (Sullivan and Wang, 2024; Li et al., 2025). However, transnational individuals differ in the forms, duration, and extent of their engagement with the societies in which they live, and the experiential foundations they develop and the ways in which these experiences are expressed through media therefore also vary considerably. Compared with short-term travelers or transnational creators in general, international students enter new social environments through educational mobility and develop sustained social contact over extended periods of study and everyday life. Their transnational experiences are typically accompanied by cross-cultural adaptation and the reconfiguration of social relationships, while digital media provide an important space for maintaining transnational connections and expressing personal experiences (Rui and Wang, 2015; Pang, 2020). At the same time, international students' understandings of the societies in which they study do not necessarily converge as a result of their study-abroad experiences; their evaluations remain shaped by their home-country cultures, personal experiences, and specific contexts (Bislev, 2017; Mulvey, 2020; Song and Xia, 2021). International students' digital narratives are therefore better understood as personalized expressions grounded in sustained experiences of transnational life, providing a distinctive setting for examining how firsthand experiences enter transnational digital communication. In this study, such content—formed through actual experiences of transnational life, organized through digital media, and expressed publicly—is conceptualized as transnational experiential narratives, which serve as the object of analysis for examining how individual experiences enter transnational digital communication and subsequently develop through audience processes.

Existing research on the digital expression of transnational individuals has gradually expanded from communicative actors and content production to the audience level. Previous studies have largely examined how foreign creators represent other societies, organize narrative frames, and participate in constructing national and cultural meanings (Cho-Li et al., 2025; Li et al., 2025; Sullivan and Wang, 2024). Audience comments have also begun to be used to investigate how users understand and evaluate cross-cultural content (Whyke et al., 2022; Jiao et al., 2022). Nevertheless, once the analytical focus shifts from content presentation to audience responses, two levels of analysis warrant further development. First, platform comments are commonly used to identify overall themes, attitudes, or semantic structures, whereas relationships between specific top-level comments and subsequent replies are less often treated as a primary unit of analysis. This makes it possible to identify what kinds of responses audiences form, but more difficult to observe how those responses are retained or shifted in subsequent exchanges. Digital discourse research suggests that subsequent responses may re-use, reinterpret, or reframe prior discourse in new interactional contexts (Perrino, 2017; Weizman, 2023). However, how specific audience responses are retained or shifted between comments and replies in the context of transnational experiential narratives remains underexplored. Second, different audience responses do not necessarily operate independently. Relational engagement, information seeking, experiential evaluation, and broader social interpretation may coexist within the comment space surrounding the same video. Because complex outcomes may be associated with the joint presence of multiple conditions and because different combinations may be associated with similar outcomes, the relationship between audience response structures and platform communication performance may exhibit configurational rather than single-factor characteristics (Fiss, 2011; Misangyi et al., 2017). Addressing these two gaps extends the analysis of audience responses from thematic identification to interactional processes of meaning-making and from individual response themes to the combined structures of multiple responses.

Against this background, this study focuses on the transnational digital narratives shared on YouTube by international students in China and the interactions that develop in their comment spaces. Beginning with the thematic structure of audience responses, it further examines thematic changes in comment interactions and analyzes the configurational relationships between different response themes and platform communication performance. First, BERTopic is used to identify audience response themes and their structure from naturally occurring comments. The relationships between comments and replies are then analyzed to examine the retention and transition of different themes during interaction. Finally, audience response themes are aggregated to the video level, and fuzzy-set qualitative comparative analysis (fsQCA) is employed to analyze the configurational relationships between different combinations of themes and high platform communication performance. This analytical framework extends the study of audience responses from thematic content to subsequent interaction and further connects the thematic structure of responses with video-level platform performance. In doing so, it offers an integrated empirical framework for understanding how transnational experiential narratives are interpreted, how response focuses shift in subsequent interaction, and how different response structures are associated with communication performance on digital platforms.

Accordingly, this study addresses the following research questions:

RQ1: What audience response themes and structural characteristics emerge from YouTube narratives about studying and living in China produced by international students in China?

RQ2: How are these audience response themes retained or shifted between top-level comments and associated replies?

RQ3: How are different audience response themes configurationally associated with higher composite communication performance?

## 2 Literature review and theoretical framework

### 2.1 From digital media practices to transnational experiential narratives: international students as cultural intermediaries

Early discussions of international students and digital media focused primarily on cross-cultural adaptation, the maintenance of social relationships, and transnational connections. Social media enable international students to maintain existing relationships after moving into a new social environment while also establishing new social networks, and their patterns of media use are closely associated with cross-cultural adaptation and social support (Rui and Wang, 2015; Pang, 2020). This line of research has gradually demonstrated that digital media are not auxiliary tools external to international students' experiences abroad. Rather, they are embedded in students' learning, social interaction, and everyday practices. Their platform-based expressions are therefore grounded distinctly in the practices of everyday life (Zhou and Yin, 2025).

The transnational mobility of international students has also long been discussed within the frameworks of educational diplomacy, public diplomacy, and soft power, with their educational experiences regarded as potentially influencing transnational perceptions and social relations. Empirical studies, however, show that student status does not correspond straightforwardly to particular communication outcomes (Lomer, 2017; Ayhan et al., 2022). Bislev (2017) points out that the relationship between international students and national soft power is shaped by factors such as their actual study-abroad experiences and individual judgments. Mulvey (2020) further emphasizes the role of student agency in the relationship between international education and public diplomacy, demonstrating that individuals' understandings of their host societies and their subsequent actions are highly context dependent. Discussions of policies and practices concerning international students likewise show that institutional expectations regarding students' roles in cross-cultural exchange cannot fully capture their actual social positions and media practices (Mulvey and Lo, 2021; Dwyer et al., 2021). Taken together, these findings suggest that international students' communicative practices cannot be understood solely in terms of predetermined institutional functions. Attention must also be directed to their concrete lived experiences and the personal expressions arising from those experiences.

Digital platforms have further brought international students' experiences of transnational life into the sphere of public communication. Research on transnational social media creators has observed that individuals' social positions, cross-cultural experiences, and modes of expression influence how foreign societies are selected, organized, and represented. Personal experience can thereby participate in the production and negotiation of cross-cultural meaning (Cho-Li et al., 2025; Li et al., 2025; Sullivan and Wang, 2024). For international students, campus experiences, learning processes, and everyday social encounters originate within specific study-abroad contexts. Once these experiences are organized and publicly shared through videos, text, and other forms, however, they become media content that other platform users can access and interpret. The perspective of the cultural intermediary provides a theoretical basis for understanding this transformation from lived experience into digital expression. Research on cultural intermediaries emphasizes that actors situated between cultural production, circulation, and reception do not simply transmit existing content. Instead, they participate in selecting, organizing, interpreting, and re-presenting cultural materials, while their specific practices are also shaped by their social positions and contextual conditions (Hutchinson, 2020; van Driel and Dumitrica, 2021; Colucci and Pedroni, 2022). From this perspective, international students' digital practices involve more than making personal experiences public. They also entail selecting and narratively organizing experiences of transnational life, thereby transforming experiences originally embedded in specific contexts of everyday life into digital content capable of entering transnational communication.

This theoretical perspective has increasingly been applied to digital creators with cross-cultural experience. Pan (2026) situates international students' short-video practices within processes of cultural intermediation, emphasizing the role of cross-cultural experience and language competence in interpreting and translating experiences of the host society. Research on foreign digital creators likewise demonstrates that creators' experiential positions influence both how foreign societies are represented and how audiences understand the resulting content (Li et al., 2025). These studies extend discussions of cultural intermediation to the digital media practices of transnational individuals and provide an empirical basis for understanding how international students transform firsthand experiences into digital expressions addressed to platform audiences. Building on this line of research, the present study defines personalized content that is grounded in sustained experiences of transnational life, organized through digital media, and expressed publicly as transnational experiential narratives. In this study, transnational experiential narratives refer to the experiential digital content under analysis, whereas cultural intermediation provides the analytical lens for understanding how international students select, organize, and translate such content from their situated experiential positions. This concept establishes a basic framework for understanding international students' transnational digital expression while directing the research focus toward the processes through which audiences understand, respond to, and interact with these narratives after they enter digital platforms.

### 2.2 From audience responses to interactional recontextualization: meaning-making in the comment space

The development of digital platforms has gradually shifted audience research from information reception toward participatory meaning-making. The concept of participatory culture emphasizes that ordinary users can continue to intervene in the circulation and interpretation of media content through commenting, sharing, and content re-creation, thereby rendering the previously clearer boundaries between communicators and audiences increasingly open (Mannell and Smith, 2022; Li and Pang, 2024). This perspective has encouraged social media research to engage more directly with naturally occurring user discourse, and comment texts have consequently been used extensively to identify the issues that concern audiences, their evaluative orientations, and their structures of meaning (Reimer et al., 2023). Research on cross-cultural digital communication also demonstrates that audiences interpret and negotiate information provided by creators by drawing on their own experiences and prior judgments; their responses are not simply repetitions of the original narratives (Li et al., 2023; Zhang et al., 2024). The comment space therefore not only presents audiences' actual responses to communicated content but also constitutes an important site for observing how transnational content is further understood and discussed.

Audience participation in platform comments, however, does not consist of mutually isolated expressions of opinion. Comments and replies instead form sequentially connected interactional relationships (Byun et al., 2023; Jakob et al., 2024). From the perspective of discursive contextuality, the meaning of a reply must be understood in relation to the preceding discourse to which it responds, the target of the response, and the specific communicative context. In other words, subsequent expressions are intrinsically connected to the context of the preceding discussion (Weizman, 2023). On this basis, recontextualization further reveals how meaning changes within such interactional relationships: when existing discourse enters a new response context, it may be reselected, reinterpreted, and reframed, thereby acquiring a direction of meaning different from that found in its original context (Gruber et al., 2023). Whereas cultural intermediation primarily concerns creators' selection, organization, and translation of transnational experience, interactional recontextualization concerns how existing expressions are re-invoked, interpreted, and reframed when they enter subsequent interactional contexts. This mechanism is also present in digital media interactions. Perrino (2017), for example, demonstrates that preceding discourse can be invoked again in subsequent comments and incorporated into new frames of discussion. Existing research on transnational digital communication, however, has primarily identified the issues and evaluations formed by audiences from comments considered in aggregate. It has paid less attention to the processes through which these responses are retained within specific comment–reply relationships or transition toward other themes.

Furthermore, changes in meaning across comments and replies do not occur independently of their specific content environments. When large numbers of audience members participate continuously in discussions surrounding the same video, different responses—including relational interaction, information seeking, experiential evaluation, and broader social comparison—may coexist and jointly constitute the overall response structure of that video's comment space (Wang and Luo, 2023). The relationship between audience responses and platform communication performance is consequently difficult to understand solely in terms of the independent effect of any single theme (Ma et al., 2025). A configurational perspective emphasizes that complex outcomes are often related to the joint structure of multiple conditions and that different combinations of conditions may be associated with similar outcomes, thereby exhibiting configurational properties such as conjunctural causation and equifinality (Furnari et al., 2021). This perspective offers an appropriate analytical approach for further understanding the diverse responses found in platform comment spaces: audience discussions surrounding different videos do not merely represent the simple accumulation of several response themes but may instead exhibit distinct configurational structures of themes. Accordingly, after examining how audience responses are retained or shifted between comments and replies, these responses must also be aggregated to the video level to analyze the configurational associations between different combinations of themes and high platform communication performance. The former focuses on shifts in response focus during interaction, whereas the latter examines video-level thematic configurations; together, they capture audience processes at two distinct analytical levels.

## 3 Research design and methods

### 3.1 Research platform and sample construction

Considering the circulation scope and interactional features of transnational digital narratives, this study selects YouTube as the research platform. As a video platform with a global user base, YouTube allows individual creators from different countries and regions to continuously publish everyday-life content and circulate it across borders. Its public comment and reply structure also provides naturally occurring empirical material for observing audience responses and interactions after content enters audience spaces. For international students in China, videos related to study, campus life, and everyday living can transform sustained transnational living experience into public digital narratives addressed to audiences in different regions, making YouTube well suited to the study of transnational experiential narratives, audience responses, and subsequent interaction.

This study focused on international student creators with verifiable study experience in China and on YouTube videos directly related to their study and everyday life in China. Platform searches were conducted intensively in August 2026, and the final sample confirmation and data retrieval were completed on August 10, 2026. Searches centered on English keywords such as study abroad in China, international student in China vlog, student life at university in China, China campus vlog, and exchange student China vlog, and were further expanded by combining degree level, city, university names, and contexts such as study, accommodation, language, and campus life.

To expand retrieval coverage as much as possible and reduce omissions caused by reliance on a single keyword, sample construction followed a continuous procedure of multiple rounds of platform search, automatic preliminary screening, manual verification, and supplementary checking. A pilot search using eight keyword groups yielded 231 candidate videos after deduplication. A second search using 35 expanded keyword groups yielded 1,492 deduplicated candidate videos. A further supplementary search using 45 additional keyword groups broadened retrieval coverage and yielded 2,360 candidate videos in that round. After the latter two rounds were merged and deduplicated by video ID, a candidate pool of 3,285 videos involving 1,856 different YouTube channels was formed. After automatic priority screening based on topical relevance and content format, 1,759 videos involving 1,001 channels were retained for manual verification. The search and screening records show that the formal sample was not generated directly from a single platform search result, but was gradually determined through expanded retrieval and manual identity verification.

Manual verification focused on creator identity, Chinese context, content relevance, and data availability. Creators had to be confirmable through channel descriptions, self-introductions, sustained published content, or related videos as having experience of university study, degree study, or exchange study in China. Videos had to be primarily situated in China and substantively involve course learning, campus life, accommodation, social interaction, language learning, or other everyday experiences of living in China. Videos were excluded when international student identity could not be verified, when they lacked substantive relevance to study and life in China, when they mainly consisted of short-video or fragmented content, or when comments or key platform data could not be retrieved normally.

Among the 1,759 manually checked videos, the first round produced 71 direct inclusions, 1,382 videos reserved for further verification, and 306 exclusions. After a second round of creator-identity and video-context verification, the first formal set comprised 130 videos from 73 channels. To increase the coverage of comment-interaction materials, videos among the remaining reserved cases that had high topical relevance and at least 100 platform comments were then prioritized for additional review. After creator identity and content relevance were rechecked, 55 videos from 46 previously unrepresented channels were added. The final formal sample comprised 185 videos from 119 different channels. This supplementary checking increased the visibility of videos with richer comment interaction in the final sample. The study therefore uses a purposive platform-content sample rather than a probability sample of all YouTube videos by international students in China. This strategy is consistent with the analytical aims of examining naturally occurring comment themes, comment–reply interaction, and video-level configurational relationships, but the study does not use it to estimate the population distribution of all such videos or all audience responses on YouTube. Further details are presented in Table 1.

**Table 1:** Video search, screening, and formal sample construction.

Stage	Operation and screening rule	Result
Pilot search	Eight core keyword groups; up to 50 videos retrieved per group	231 videos after deduplication
Expanded search	Thirty-five keyword groups; up to two pages of results per group	1,492 videos after deduplication
Supplementary search	Forty-five supplementary keyword groups used to expand retrieval coverage	2,360 videos in this round
Candidate-pool merging	Expanded-search and supplementary-search results merged and deduplicated by video ID	3,285 videos and 1,856 channels
Automatic priority screening	Priority given to topically relevant videos that were not short-form content	1,759 videos and 1,001 channels entered manual verification
First manual verification	Identity, account type, and content relevance checked	INCLUDE 71; RESERVE 1,382; EXCLUDE 306
Second manual verification	RESERVE videos and videos with insufficient identity information further verified	First formal sample of 130 videos from 73 channels
Supplementary manual verification	Additional review of high-relevance RESERVE videos with comment count >=100	55 videos added from 46 new channels
Final formal sample	Identity, content, and data availability reconfirmed and deduplicated	185 videos from 119 channels/creator accounts

### 3.2 Data collection and text processing

#### 3.2.1 Data collection

Python 3.13.14 and the YouTube Data API v3 were used to collect publicly available information and comment data for the sampled videos. Final data retrieval and merging were completed on August 10, 2026. At the video level, the records included video ID, channel information, publication date, view count, like count, comment count, channel subscriber count, and a unified observation time. At the comment level, the records included comment type, unique comment ID, comment text, publication time, like count, and the parent–child relationship between top-level comments and replies. The program retrieved top-level comments and all available replies while preserving comment IDs for subsequent deduplication and pair verification. A total of 39,810 original merged comment-and-reply records were obtained from the 185 videos in the final sample.

#### 3.2.2 Text preprocessing and language filtering

Text preprocessing followed a relatively conservative principle in order to preserve the colloquial expressions, abbreviations, and natural interactional features of YouTube comments as far as possible. First, the 39,810 original records were subjected to basic cleaning. Only records with no analyzable semantic content or records clearly constituting data noise were removed, including 1,204 pure emoji or symbol texts, 917 duplicates, 63 obvious spam records, 42 pure URLs, and 14 empty texts. In total, 2,240 records were removed, leaving 37,570 valid comments and replies. For texts entering language identification, the original comment content was retained; the analysis text used for modeling removed only URLs, emoji, and other non-semantic symbols, without excessive stemming or mechanical deletion of ordinary words with semantic functions.

Because BERTopic embedding representation, topic clustering, and subsequent comparison of top-level comments and replies all require a relatively consistent language-semantic space, the 37,570 valid texts were further filtered to construct an English corpus and reduce additional measurement differences caused by multilingual mixing. The screening combined writing-system features, English lexical features, and automated language-identification results to distinguish clearly English, clearly non-English, and boundary cases. Texts that were too short, had insufficient language confidence, or showed obvious mixed-language features were handled conservatively. This stage yielded 29,213 English candidate texts, 2,344 borderline texts, and 6,013 non-English texts or texts with no valid alphabetic content. Borderline and non-English texts were retained in the audit data but were not included in the formal topic model.

On the basis of the 29,213 English candidate interaction texts, the formal analytical corpus was further constructed by combining language-quality screening, comment type, and creator-identity information. BERTopic topic identification used only 14,815 high-confidence English top-level comments and excluded top-level comments posted by creators themselves, so that RQ1 would primarily reflect external responses formed after video publication. Replies were screened using the same language-quality standards, resulting in 8,728 high-confidence English replies. Among these, 7,682 replies could be linked effectively to the 14,815 eligible top-level comments and served as the initial pairing range for the subsequent analysis of thematic correspondence between comments and replies. Of the 185 videos in the final sample, 182 contained at least one eligible English top-level comment usable for topic aggregation. Further details are presented in Table 2.

**Table 2:** Comment-Corpus Preprocessing and Construction of the Formal Analytical Corpus.

Processing stage	Stage input	Exclusion or processing rule	Stage output	Subsequent use
Raw data collection	185 videos	Top-level comments and all available replies collected; parent-child relationships and comment IDs retained	39,810 original merged records	Raw interaction corpus
Basic text cleaning	39,810 records	Removed 1,204 pure emoji/symbol texts, 917 duplicates, 63 spam records, 42 pure URLs, and 14 empty texts	37,570 records	Language filtering
Language classification	37,570 records	Distinguished English, Borderline, and Non-English/no valid alphabetic content	English 29,213; Borderline 2,344; Non-English 6,013	Further verification of English corpus
Formal top-level comments	English candidate corpus	High-confidence English; top-level comments; creator's own content excluded	14,815 comments	BERTopic identification and video-level aggregation
Formal English replies	English candidate corpus	High-confidence replies retained using the same language-quality standard; creator replies marked but not removed	8,728 replies	Reply-theme analysis
Linkable replies	8,728 replies	Parent comment belongs to the 14,815 eligible top-level comments	7,682 replies	Initial RQ2 comment-reply pairs
Videos with valid themes	185 videos in the final sample	At least one eligible English top-level comment usable for topic aggregation	182 videos	Video-level thematic analysis and candidate fsQCA sample

### 3.3 Topic identification and variable construction

BERTopic (version 0.17.4) was applied to the 14,815 eligible English top-level comments for topic identification. BERTopic obtains contextual semantic representations of texts through pretrained language models and combines dimensionality reduction, density clustering, and topic-word representation to identify latent thematic structures in short texts. It is therefore suitable for YouTube comments, which are short, colloquial, and heterogeneous in expression (Grootendorst, 2022; Doogan et al., 2023; Nanayakkara and Thennakoon, 2024). The formal analysis used sentence-transformers/all-MiniLM-L6-v2 to generate text embeddings and then applied UMAP for dimensionality reduction with n_neighbors = 15, n_components = 5, min_dist = 0.0, metric = cosine, and random seed fixed at 42. HDBSCAN was then used for density clustering with min_cluster_size = 100, min_samples = 5, metric = euclidean, and cluster_selection_method = eom. Topic-word representation used CountVectorizer with unigrams and bigrams (n-gram range = 1–2), English stop words, min_df = 3, and max_df = 0.95, together with c-TF-IDF and KeyBERTInspired to extract topic keywords. Fifteen main keywords were retained for each topic. The number of topics was not predetermined. The model ultimately identified 39 valid micro-topics (Topic 0–38). The substantive interpretability of the 39 micro-topics was assessed mainly according to the semantic consistency of high-weight keywords, the internal semantic consistency of representative comments, and substantive distinguishability across topics. After manual semantic review, all 39 micro-topics had identifiable and relatively clear discussion focuses and were therefore included in subsequent higher-order thematic aggregation. Another 5,746 top-level comments were identified by HDBSCAN as outliers (Topic = −1), accounting for 38.79% of the eligible top-level comments; these texts were not forcibly reclassified at the topic-modeling stage.

On the basis of micro-topic identification, the study further summarized the main objects of discussion and response content of the 39 micro-topics by considering each topic's high-weight keywords, representative comments, and overall semantics, and used this process to form a candidate hierarchical thematic framework. Unified coding criteria were then developed following established recommendations for assessing intercoder reliability (Lombard et al., 2002). Two researchers independently determined the subtheme and overarching-theme assignment of all 39 micro-topics without seeing each other's coding results. Agreement for overarching themes was 92.3%, with Cohen's kappa = 0.90; agreement for subthemes was 84.6%, with kappa = 0.82. For micro-topics with disagreements, the two researchers reviewed topic definitions, keywords, and representative comments and reached consensus through discussion. A fixed mapping among 39 micro-topics, 15 subthemes, and six overarching audience response themes was ultimately established. Intercoder agreement here was used mainly to test the reliability of researchers' higher-order aggregation of machine-generated micro-topics, rather than to represent classification accuracy at the level of individual comments.

Because the subsequent comment-and-reply analysis required individual texts to be mapped onto the six-theme framework, the study further conducted independent document-level manual validation. Stratified samples of 250 top-level comments and 180 replies were drawn from the formal corpus, with sampling taking into account model-predicted topics, HDBSCAN clustering status, and classification confidence. Coders independently judged each text's theme without seeing model topic labels or probability information. Texts with insufficient information, clear semantic boundary problems, or no reasonable fit within the six themes were not forcibly classified. Manual coding showed that 240 of the 250 top-level comments and 151 of the 180 replies could be clearly assigned to the six overarching themes; the remaining texts were treated separately as indeterminate cases. To obtain continuous thematic information for individual texts across the six overarching themes, BERTopic's approximate_distribution was then used to estimate each text's soft distribution over the 39 micro-topics, with window = 8, stride = 4, min_similarity = 0.0, and batch_size = 500. According to the fixed 39-to-6 mapping, micro-topic weights belonging to the same overarching theme were summed and normalized by the total micro-topic weight of the text, thereby producing the relative salience of the six overarching themes.

For dominant-theme assignment, texts already assigned by HDBSCAN to Topic 0–38 were directly assigned to an overarching theme according to the fixed 39-to-6 mapping. For outlier texts with Topic = −1, the difference between the highest and second-highest salience values among the six overarching themes was compared. To avoid forcing classification of texts with unclear thematic boundaries, four difference thresholds, 0.03, 0.05, 0.08, and 0.10, were compared, and the validation texts that manual coders could clearly assign to the six themes were stratified by theme into a 60% development sample and a 40% holdout sample for checking. After balancing classification agreement and sample retention, 0.05 was selected as the formal criterion: when the difference between the highest and second-highest salience values was at least 0.05, the theme corresponding to the highest value was assigned as the dominant theme; when the difference was below 0.05, or when no valid soft distribution existed, the text was retained as indeterminate and excluded from subsequent dominant-theme correspondence analysis.

Under these assignment rules, the dominant-theme assignment coverage among validation texts that manual coders could clearly classify into the six overarching themes was 78.8% for top-level comments. Among top-level comments with clear model assignments, agreement between the model and manual coding was 62.4%, Cohen's kappa = 0.546, and Macro-F1 = 0.606. For replies, dominant-theme assignment coverage was 78.1%; among replies with clear model assignments, agreement between the model and manual coding was 66.9%, Cohen's kappa = 0.595, and Macro-F1 = 0.579. These results indicate that the study did not classify all texts unconditionally by maximum probability, but instead established explicit rejection rules for outlier texts and texts with semantic boundary problems while maintaining a certain level of corpus coverage. The document-level assignment results were then used for comment–reply thematic correspondence and transition analysis.

### 3.4 Thematic correspondence and transition between comments and replies

To ensure that top-level comments and replies could be compared within the same thematic space, the study did not train an independent topic model for replies. Instead, the 8,728 eligible English replies were mapped onto the BERTopic topic space established from top-level comments and were assigned dominant themes according to the six-theme framework and document-level assignment rules. Using a shared thematic space facilitates direct comparison of thematic correspondence between parent comments and replies, but it may also constrain themes that appear only in reply texts. To reduce this risk, the study explicitly limits the scope of interpretation and directly validates document-level classification using 180 independently blind-coded replies.

Among the 8,728 eligible English replies, 7,682 could be linked effectively to eligible top-level comments. According to the final theme-assignment rules, 6,342 parent-comment instances and 6,178 reply instances in these pairs received clear themes. When both sides of a pair received a clear dominant theme within the six-theme framework, 5,211 valid top-level-comment–associated-reply pairs were formed, accounting for 67.8% of all linkable replies. Of these, 4,789 were ordinary user replies and 422 were creator replies, accounting for 8.1%. To fully reflect the associated-reply structure in the sample, both ordinary user replies and creator replies were included in the overall analysis, and replier type (ordinary user vs. creator) was retained for subsequent analysis. If a top-level comment and its associated reply had the same dominant theme, the pair was coded as thematic retention; if their dominant themes differed, it was coded as thematic transition. The study then calculated the six-theme parent-comment-by-reply matrix, theme-specific retention rates, major cross-theme transition directions, and net thematic changes. These indicators describe only the retention or change of dominant issue focus between top-level comments and their associated replies. They do not equate thematic transition itself with full discursive recontextualization, nor do they reconstruct complete multiround conversational development. Because comment–reply pairs are nested within the same video and even within the same discussion thread, this section uses descriptive structural analysis and does not treat the 5,211 pairs as mutually independent cases for statistical inference.

### 3.5 Measurement of communication performance and configurational analysis

#### 3.5.1 Construction of video-level thematic conditions

In the video-level configurational analysis, this study uses the six audience response themes as condition variables. The six-dimensional relative thematic salience at the single-comment level is used to characterize the relative composition of different themes within a comment. Because the dimensions are relatively dependent, these values are not directly used as condition variables for the video-level configurational analysis. Therefore, based on the 39 micro-topic weights output by BERTopic, this study calculates the activation levels of the six themes separately and further aggregates them to the video level, thereby forming the continuous condition variables used in the fsQCA analysis.

Let *p*_*ij*_ denote the soft weight of the *i*-th top-level comment on the *j*-th micro-topic, and let *M*_*k*_ denote the set of micro-topics belonging to the *k*-th overarching theme. Because the six overarching themes contain different numbers of micro-topics, this study uses the root mean square (RMS) to aggregate the micro-topic weights under the same overarching theme:


$$\begin{array}{l} A_{ik}=\sqrt{\left(\frac{1}{\left|M_{k}\right|}\right)\underset{j\in M_{k}}{\sum }p_{ij}^{2}} \end{array}$$ (1)

where *A*_*ik*_ denotes the activation value of the *i*-th comment on theme *k*, and |*M*_*k*_| denotes the number of micro-topics included in that theme. This calculation retains the overall activation level of the comment on the corresponding thematic dimension while correcting for differences in the number of micro-topics contained in different overarching themes. On this basis, the comment-level theme activation values are aggregated by video. Let *N*_*v*_ denote the number of eligible English top-level comments contained in the *v*-th video. The condition value of that video on theme *k* is:


$$\begin{array}{l} X_{vk}=\left(\frac{1}{N_{v}}\right)\overset{N_{v}}{\underset{i=1}{\sum }}A_{ik} \end{array}$$ (2)

where *X*_*vk*_ denotes the average activation level of the *v*-th video on theme *k*. Through the above procedure, each video obtains six continuous thematic conditions for the subsequent fsQCA configurational analysis.

#### 3.5.2 Outcome variable for communication performance

Using the video as the unit of analysis, platform communication performance was constructed from publicly available YouTube data on views, likes, publication time, and the unified data-retrieval time. Because sampled videos differed in time online and cumulative exposure scale, direct comparisons of views and likes may be affected by publication time and existing exposure. The study therefore measured communication performance along two dimensions: Reach and Approval. Reach was measured as average daily views, calculated by dividing cumulative views by the number of days from publication to the unified observation date. Approval was measured as likes per 1,000 views.

In the main sample, average daily views and likes per 1,000 views were transformed using ln(1+X) to reduce the influence of extreme values and right-skewed distributions, and then standardized as Z scores. Because there was no sufficient theoretical or empirical basis for assigning different weights to the two dimensions, the study constructed a composite communication performance index using equal weights:


$$\begin{array}{c} \text{CompositeY}=0.5\times Z[ln(1+\text{views/day})]+0.5\times Z[ln(1\\ \;+\text{likes}/1,000\text{ views})] \end{array}$$ (3)

This composite index jointly represents the two distinct outcome dimensions of communication reach and audience approval rather than measuring a single latent construct. In the main sample, the Pearson correlation between the two components was −0.103 and the Spearman correlation was −0.063, indicating that the two dimensions reflect strongly distinct aspects of communication performance. Therefore, in addition to using Composite Y as the main outcome variable, the study further used Reach and Approval separately as alternative outcome variables in sensitivity analysis to assess whether the configurational findings depended on the operationalization of the composite index. Comment count was not included in the outcome variable in order to avoid mechanical overlap with thematic conditions constructed from comment texts.

#### 3.5.3 Analytical sample and fuzzy-set calibration

Of the 185 videos in the final sample, 182 had valid thematic data, and 154 contained at least 10 eligible English top-level comments. To reduce measurement fluctuation in video-level thematic means when comment counts were too small, the main analysis used at least 10 eligible English top-level comments as the minimum threshold. Another 12 videos meeting this threshold had zero recorded likes; because current platform data cannot reliably distinguish true zero likes from unavailable or hidden like records, these cases were not included in the Approval and Composite Y analyses. The final main fsQCA sample therefore comprised 142 videos from 99 different creators. In the main sample, a single creator contributed at most five videos, and the five most represented creators together accounted for 16.2% of the sample.

Direct calibration was used to transform the six thematic conditions and outcome variables into fuzzy-set membership scores between 0 and 1 (Ragin, 2008; Schneider and Wagemann, 2012). Because the BERTopic-derived RMS theme activation values and platform communication performance indicators lack externally validated substantive thresholds, the 10th, 50th, and 90th percentiles of the empirical distributions across the 142 cases were used as the anchors for full non-membership, the crossover point, and full membership in the main analysis, corresponding to membership scores of 0.05, 0.50, and 0.95, and continuous fuzzy-set membership scores were obtained through log-odds transformation. The 10th percentile indicates clear non-membership in the set of high salience for a given theme or high communication performance; the 50th percentile indicates the point of maximum ambiguity; and the 90th percentile indicates clear membership in the corresponding set. As a calibration sensitivity test, P05/P50/P95 anchors were also used. No cases fell exactly at 0.50 in either the main or alternative calibration, so no manual adjustment of crossover points was required. The specific anchors are presented in Table 3.

**Table 3:** Calibration Anchors for the fsQCA Variables.

Condition/outcome	P10 full non-membership	P50 crossover	P90 full membership	Alternative P05	Alternative P95
Relational and affective engagement	0.022496	0.029862	0.047127	0.020563	0.056773
Educational pathway exploration	0.025065	0.033416	0.044643	0.022979	0.047655
Study-abroad experiences in China	0.027609	0.034323	0.046090	0.025879	0.048558
Language learning and academic adaptation	0.020806	0.027898	0.038033	0.019197	0.040258
Study-abroad risk appraisal	0.020234	0.029971	0.039242	0.017063	0.042147
National-frame recontextualization	0.018840	0.024351	0.031466	0.017697	0.034243
Composite Y	−0.868018	0.032120	0.850506	−1.037754	1.136106
Reach	1.492950	3.326894	5.727041	1.316840	6.277176
Approval	2.753276	3.348200	3.894147	2.601986	4.053327

## 4 Results

### 4.1 Thematic structure and semantic features of audience responses

The topic analysis ultimately produced six audience response themes: Relational and Affective Engagement, Educational Pathway Exploration, Study-Abroad Experiences in China, Language Learning and Academic Adaptation, Study-Abroad Risk Appraisal, and National-Frame Recontextualization. Their subthemes, semantic features, and representative comments are shown in Table 4.

**Table 4:** Thematic Structure of Audience Responses.

Overarching theme	Subtheme	Theme explanation	Representative comment (excerpt)
Relational and affective engagement	Positive evaluation and content approval	Recognition expressed through praise, thanks, and positive evaluation of video content, reflecting positive affective engagement with the narrative content.	this was very interesting thanks for sharing
	Congratulations, support, and encouragement	Congratulations, encouragement, and blessings around graduation, academic progress, personal growth, and creator effort, reflecting sustained supportive responses.	Congratulations to you! Well done! God bless you!
	Individual attention and parasocial closeness	The response focus shifts from the video topic to the creator's appearance, personality, and personal characteristics, reflecting sustained attention to the creator and parasocial closeness.	You look so beautiful! I love that red lipstick
Educational pathway exploration	University choice and campus/course information	Questions or comparisons concerning universities, programs, campus environment, and study experience, treating creator experience as an information source for potential educational environments.	Sis in which university are u studying
	Scholarship, application, and contact consultation	Concrete operational information sought about scholarships, eligibility, admission procedures, and contact channels, turning video experience into practical application resources.	I want to apply... what should I do now to come for bachelor there on fully funded scholarship...
	Academic stage and future-development planning	Creator experience linked to the audience's own academic stage, major choice, and future educational development, showing forward-looking educational planning.	Hopefully I am in med school next year
Study-abroad experiences in China	Life in China and overall study-abroad experience	Creator experience used to understand the overall state of studying, living, and traveling in China and to form an integrated view of study life in China.	Love your video... your experience good/bad as a foreign student...
	Campus environment, everyday study, and alumni memory	Discussion of campus environment, dining halls, everyday coursework, and university life, including alumni memories and comparisons based on prior campus experience.	I grew up in Nanjing. Nanjing University is actually my alma mater too...
	Dormitories, accommodation costs, and living conditions	Questions, comparisons, and evaluations concerning dormitory space, room type, rent, accommodation fees, and living facilities.	How much does it cost for the hostel/dorm fee on monthly basis?
Language learning and academic adaptation	Chinese-language learning and language competence	Attention to Chinese-learning methods, learning duration, fluency, and motivation, reflecting concern with the formation of cross-language competence.	How long did it take you to learn Mandarin...?
	Chinese-medium instruction and academic adaptation	Discussion of Chinese-medium instruction, differences between English- and Chinese-taught programs, classroom comprehension, and difficulties in specialized study.	I did my bachelors in Computer Science in Chinese, and it was honestly a roller coaster...
Study-abroad risk appraisal	Education quality and institutional problems for international students	Questioning international-student education quality, program design, learning returns, and student circumstances, reflecting critical appraisal of the institutional environment and education quality.	China hasn't got an education program for international students... Studying in China is a waste of time...
	Medical study and career-return risks	Focus on degree recognition, teaching quality, career prospects, and potential returns in medical and other specialized study, forming explicit risk judgments about study-abroad choices.	Any student who is thinking to go to China for MBBS... please Avoid...
National-frame recontextualization	Transnational identity, national comparison, and belonging	Personal study-abroad experience placed within frames of national development, identity belonging, and return choices, moving concrete experience into systems of national identity and transnational reference.	In the end, come to your country and work for its betterment
	Political system, governance, and ideological recontextualization	Campus and study-abroad experience extended to discussions of national governance, institutional difference, ideology, and international politics, re-embedding personal experience in national-political frames.	Capitalism or communism, it doesn't really matter as long as the government means well...

In terms of thematic content, Relational and Affective Engagement and Educational Pathway Exploration represent two relatively distinct orientations of audience response. Relational and Affective Engagement centers on evaluations of video content, congratulations and support, and sustained attention to the creator as an individual. It reflects relational responses that extend from content approval to emotional support and parasocial closeness. Educational Pathway Exploration has a more instrumental and planning-oriented character: audiences transform international students' firsthand experiences into resources for understanding universities and programs, obtaining scholarship and application information, and planning future academic development. Thus, after transnational experiential narratives enter the comment space, they can both foster relational engagement around content and creators and be further used by audiences to explore possible educational pathways.

Study-Abroad Experiences in China and Language Learning and Academic Adaptation locate audience responses more concretely in the processes of study-abroad life and learning. Study-Abroad Experiences in China mainly concerns the overall conditions of studying and living in China, campus environments, everyday coursework, and accommodation, and also includes comparisons and memories formed by audiences on the basis of their own experiences. Language Learning and Academic Adaptation focuses on Chinese-language learning, the development of language competence, Chinese-medium instruction, and problems of understanding and adaptation in specialized study. Together, these two themes show that audiences' understandings of study in China do not remain at the abstract level of a general idea of studying in China, but enter concrete contexts such as campus life, living conditions, language learning, and academic practice.

At the same time, some responses shift from concrete experience toward judgments about study-abroad consequences and external interpretive frames. Study-Abroad Risk Appraisal mainly concerns the quality of international-student education, program design, degree recognition, and career returns, reflecting audiences‘ cautious assessments of the costs, uncertainties, and potential consequences of study-abroad choices. National-Frame Recontextualization exhibits a more evident expansion of meaning: some audiences reinterpret individual study-abroad experiences within frames of national comparison, identity belonging, and return choices, while other discussions extend from campus and everyday experiences to issues of national governance, institutional difference, ideology, and international politics. International students' personal experiences in the comment space may therefore be used not only for understanding everyday life and making educational judgments, but also for re-embedding those experiences in national-level frames of reference.

### 4.2 Theme retention and transition across comments and replies

Among the 7,682 replies that could be linked to eligible top-level comments, 5,211 pairs ultimately received clear dominant themes on both the comment and reply sides. Of these, 2,747 pairs retained the original theme of the top-level comment, accounting for 52.7%, while 2,464 pairs transitioned to another theme, accounting for 47.3%. Overall, thematic continuation in associated replies was slightly higher than thematic transition, indicating that the discussion focus formed in top-level comments had a certain degree of continuity, while subsequent interaction also involved substantial issue shifts.

Retention and transition were not evenly distributed. Relational and Affective Engagement showed the strongest thematic continuity, with 75.1% of related replies retaining this theme. The retention rates of National-Frame Recontextualization and Educational Pathway Exploration also exceeded one half, at 56.9% and 55.0%, respectively. By contrast, Study-Abroad Experiences in China and Language Learning and Academic Adaptation were more likely to shift toward other themes in replies. Specifically, the main transitions from Study-Abroad Experiences in China were toward Relational and Affective Engagement and Educational Pathway Exploration, accounting for 24.7% and 20.3% of replies originating from that theme. Educational Pathway Exploration also frequently transitioned toward Relational and Affective Engagement and Study-Abroad Experiences in China, while replies originating from Language Learning and Academic Adaptation more often transitioned toward Relational and Affective Engagement and Educational Pathway Exploration. These patterns show that initial discussions centered on concrete study-abroad life and learning adaptation were relatively likely to move in associated replies toward relational interaction or educational information seeking, whereas relational responses themselves displayed stronger continuity. Although Study-Abroad Risk Appraisal was retained in the full matrix, the study does not further substantively interpret its specific transition directions because this theme showed comparatively weaker identification performance in reply-level manual validation.

Changes in the overall thematic distribution from the comment side to the reply side further illustrate this interactional direction. Relational and Affective Engagement and Educational Pathway Exploration showed net increases of 586 and 183 pairs, respectively, while Study-Abroad Experiences in China and Language Learning and Academic Adaptation showed net decreases of 472 and 123 pairs, respectively. National-Frame Recontextualization changed relatively little overall. Associated replies were therefore more concentrated in relational interaction and educational information seeking, whereas concrete study-abroad life and cross-language adaptation more often served as starting issues for subsequent discussion. It should be emphasized that thematic transition here refers only to a change in dominant issue focus between a top-level comment and its associated reply. It is not equivalent to full discursive recontextualization and cannot represent the continuous development of multiround conversations within the same discussion thread. The detailed distribution of thematic retention and transition is shown in Figure 1.

**Figure 1:**
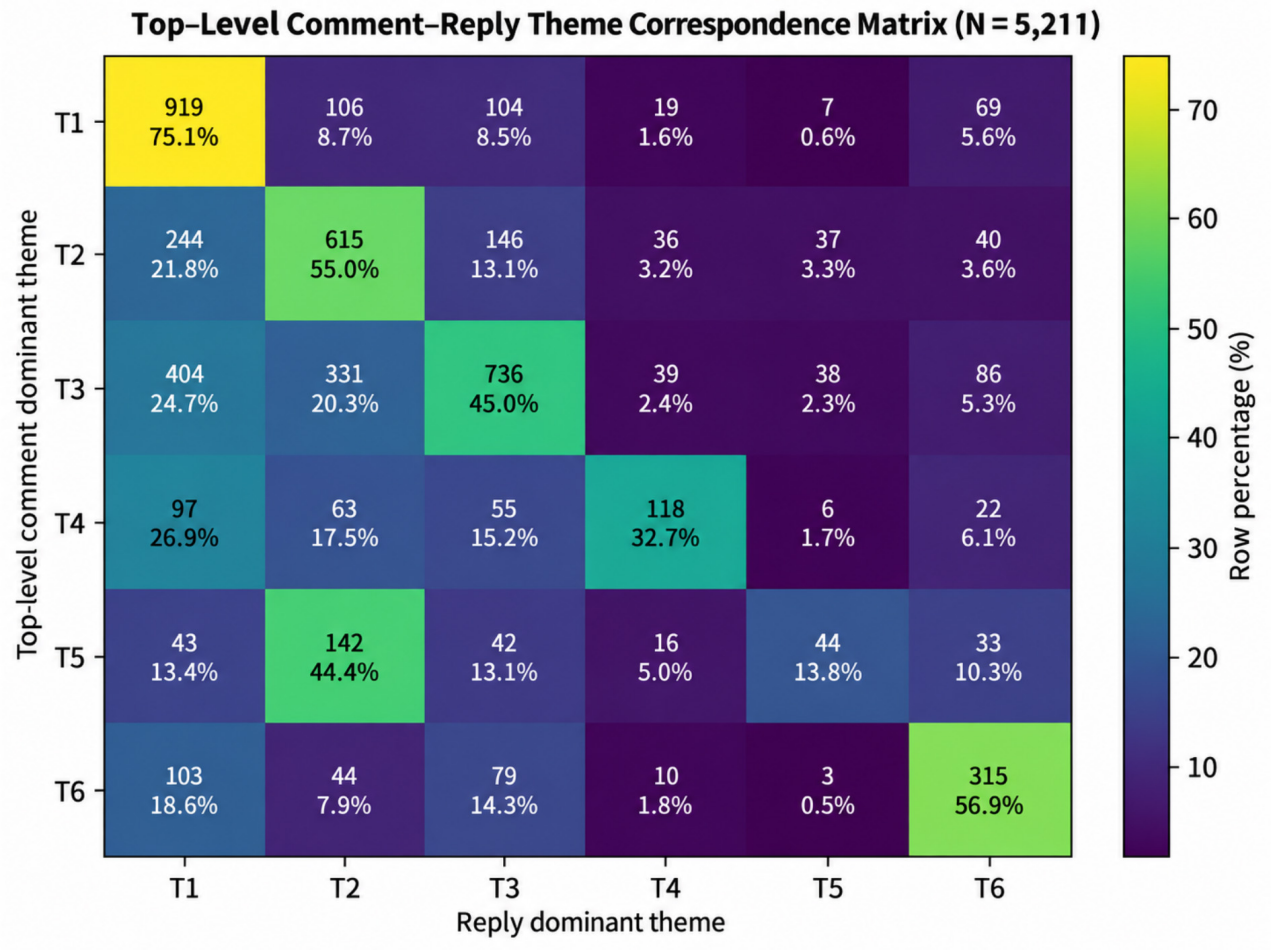
Theme retention and transition matrix between top-level comments and associated replies. The upper value in each cell indicates the number of pairs, and the lower value indicates the row percentage relative to the source theme of the top-level comment. The diagonal indicates thematic retention, and off-diagonal cells indicate dominant-theme transition. T1 = Relational and Affective Engagement; T2 = Educational Pathway Exploration; T3 = Study-Abroad Experiences in China; T4 = Language Learning and Academic Adaptation; T5 = Study-Abroad Risk Appraisal; T6 = National-Frame Recontextualization.

### 4.3 Configurations of audience response themes and high composite communication performance

#### 4.3.1 Necessity analysis

Before analyzing configurational sufficiency, the study first examined whether high or low salience of the six audience response themes constituted necessary conditions for higher composite communication performance. Using consistency of 0.90 as the criterion, the results show that no single condition reached the threshold for a necessary condition. Among them, the consistency of low salience of Study-Abroad Experiences in China was the highest, at 0.732; the consistency values of high salience of Relational and Affective Engagement and high salience of National-Frame Recontextualization were 0.710 and 0.707, respectively. Higher composite communication performance therefore does not depend on any single thematic condition, but is more likely to be associated with joint configurations of multiple audience response themes. The detailed results are shown in Table 5.

**Table 5:** Necessity Analysis for Higher Composite Communication Performance.

Condition	High-salience consistency	High-salience coverage	Low-salience consistency	Low-salience coverage
Relational and affective engagement	0.710	0.721	0.527	0.500
Educational pathway exploration	0.517	0.509	0.709	0.694
Study-abroad experiences in China	0.522	0.549	0.732	0.674
Language learning and academic adaptation	0.681	0.680	0.575	0.554
Study-abroad risk appraisal	0.555	0.527	0.669	0.680
National-frame recontextualization	0.707	0.747	0.543	0.498

#### 4.3.2 Truth table and configurational sufficiency analysis

On the basis of the necessity test, a six-condition truth table was constructed. The six thematic conditions can theoretically form 64 potential configurations. Because the main analysis included 142 video cases, the case-frequency threshold was set at 3 to reduce the influence of extremely low-frequency configurations on minimization results while preserving configurational diversity in the sample. Sufficiency analysis used 0.80 as the minimum criterion for raw consistency and further set the PRI consistency threshold at 0.75. Raw consistency assesses the extent to which a condition combination constitutes a subset of higher composite communication performance, while PRI further identifies contradictory configurations that have strong set relations with both the outcome and its negation. Therefore, only configurations meeting all three criteria of case frequency, consistency, and PRI were included in positive sufficiency minimization (Greckhamer et al., 2013).

Before solving the intermediate solution, directional expectations were set according to the substantive meanings of the themes. Relational and Affective Engagement mainly reflects content approval, support, and relational closeness, so its high salience was set as consistent with higher composite communication performance. Study-Abroad Risk Appraisal mainly involves cautious evaluations of costs, uncertainty, degree recognition, and career returns, so its low salience was set as the expected direction. For Educational Pathway Exploration, Study-Abroad Experiences in China, Language Learning and Academic Adaptation, and National-Frame Recontextualization, existing research does not provide sufficient grounds for a clear unidirectional expectation, so no high- or low-salience direction was preset.

Among the 64 potential configurations, 44 appeared in the main sample, and 17 met the threshold of at least three cases. According to the criteria described above, two empirical configurations ultimately met both the raw-consistency and PRI criteria. The two configurations were identical in the states of five conditions: Relational and Affective Engagement, Educational Pathway Exploration, Study-Abroad Experiences in China, Study-Abroad Risk Appraisal, and National-Frame Recontextualization. They differed only in Language Learning and Academic Adaptation: one showed low salience of this condition, while the other showed high salience. The two configurations contained 9 and 10 cases, with raw consistency values of 0.915 and 0.948 and PRI consistency values of 0.770 and 0.870, respectively.

Because the two positive empirical configurations differed only in Language Learning and Academic Adaptation, Boolean minimization removed this condition from the intermediate solution, indicating that either its presence or absence is compatible with the configuration. The intermediate solution ultimately identified one thematic configuration associated with higher composite communication performance: high Relational and Affective Engagement, low Educational Pathway Exploration, low Study-Abroad Experiences in China, low Study-Abroad Risk Appraisal, and high National-Frame Recontextualization co-occurred. The consistency of this configuration was 0.915, and its raw coverage was 0.366. The complex solution was identical to the intermediate solution, indicating that the same combinational structure can be obtained without further simplification through logical remainders.

To further assess the relative position of different conditions in the main configuration, the study also examined the full parsimonious solution. Parsimonious minimization produced two equally parsimonious alternative solutions. The first consisted of high Relational and Affective Engagement, low Study-Abroad Experiences in China, and high National-Frame Recontextualization, with consistency of 0.881 and raw coverage of 0.443. The second consisted of low Study-Abroad Experiences in China, low Study-Abroad Risk Appraisal, and high National-Frame Recontextualization, with consistency of 0.915 and raw coverage of 0.400. Both parsimonious solutions retained low Study-Abroad Experiences in China and high National-Frame Recontextualization, indicating that these two conditions are the most stable core conditions across different minimization solutions. Relational and Affective Engagement and Study-Abroad Risk Appraisal were retained in only one parsimonious solution each, so their core status is somewhat solution dependent. Low Educational Pathway Exploration was retained only in the intermediate solution and is more appropriately treated as a peripheral, context-specific condition within the main configuration. Accordingly, the study uses the intermediate solution as the principal basis for substantive interpretation and uses the parsimonious solutions to help identify the relative stability of different conditions. The final main configuration and its sufficiency indicators are shown in Table 6.

**Table 6:** Configurations of audience response themes associated with higher composite communication performance.

Condition variable	Configuration
Relational and affective engagement	•
Educational pathway exploration	⊗
Study-abroad experiences in China	⊗
Language learning and academic adaptation	—
Study-abroad risk appraisal	⊗
National-frame recontextualization	•
Raw coverage	0.366
Unique coverage	0.366
Consistency	0.915
Overall coverage	0.366
Overall consistency	0.915

• indicates high salience of the corresponding theme; ⊗ indicates low salience; — indicates a “don't care” condition, meaning that either its presence or absence is compatible with the configuration.

Overall, higher composite communication performance does not correspond to the prominence of any single audience response theme, but is associated with a specific combinational structure formed by multiple themes. Comparing the intermediate solution with the two equally parsimonious solutions shows that low Study-Abroad Experiences in China and high National-Frame Recontextualization are retained across different minimization schemes and are the most stable core conditions. The core status of Relational and Affective Engagement and low Study-Abroad Risk Appraisal varies across parsimonious schemes, while low Educational Pathway Exploration mainly functions as a peripheral, context-specific condition. These results reflect set-theoretic associations between combinations of audience response themes and higher composite communication performance, rather than causal effects.

#### 4.3.3 Sensitivity analysis

Because the main analysis set a minimum threshold for comment count, the study first compared videos with at least 10 eligible English top-level comments with those having fewer than 10 comments. The median total views, daily views, and channel subscribers of the former group were approximately 20,502, 27.75, and 6,855, respectively, all higher than the corresponding values of 3,969, 5.57, and 2,540 in the latter group. The median likes per 1,000 views were relatively similar between the two groups, at 27.46 and 31.22. This indicates that the comment-count threshold may retain more videos with higher exposure and larger existing audience bases, making it necessary to further examine the sensitivity of the configurational results to sample selection and other analytical settings.

Sensitivity analysis followed the principle of changing one factor at a time, examining outcome variables, sample thresholds, creator repetition, calibration anchors, and the truth-table case-frequency threshold. The results show that when Composite Y continued to be used as the outcome variable, the main structure of the principal configuration was generally retained. When the case-frequency threshold was reduced from 3 to 2, the main configuration remained unchanged. When the comment threshold was relaxed to 5, and when P05/P50/P95 alternative calibration was used, more specific configurational variants emerged that added high Language Learning and Academic Adaptation to the main configuration. After limiting each creator to at most two videos, the original main configuration was still identified, and an additional configuration also appeared, indicating that the main conditional structure retained a degree of stability after controlling for repeated creator contributions.

By contrast, substituting the outcome variable produced more evident changes. With the same 142 cases, six thematic conditions, and QCA thresholds, using Reach and Approval separately as outcome variables did not identify any sufficient configuration satisfying all prespecified criteria of case frequency, raw consistency, and PRI. In the Reach analysis, the configuration closest to the criterion had raw consistency of 0.905 but PRI of 0.7499. In the Approval analysis, the corresponding configuration had consistency of 0.906 and PRI of 0.7319. Neither reached the preset standards. Thus, the main configuration corresponds primarily to higher composite communication performance jointly constituted by communication reach and audience approval, and cannot be directly extended to the two separate outcome dimensions.

Overall, high Relational and Affective Engagement, low Educational Pathway Exploration, low Study-Abroad Risk Appraisal, and high National-Frame Recontextualization showed a high degree of recurrence across different non-outcome-variable settings, and low Study-Abroad Experiences in China was also retained in most analyses. Language Learning and Academic Adaptation functioned more often as a limiting condition that varied with sample and calibration settings. The main configuration therefore exhibits a degree of structural stability across sample thresholds, creator repetition, calibration methods, and case-frequency settings, but is more sensitive to the specific operationalization of the outcome variable. The detailed results are shown in Table 7.

**Table 7:** Sensitivity tests of the configurational results.

Analytical Setting	*N*	Main result	Consistency	Coverage	PRI	Comparison with main analysis
Main analysis	142	Main configuration C1	0.915	0.366	0.807	Baseline
Reach as outcome variable	142	No sufficient configuration satisfying all prespecified criteria identified	—	—	—	Sensitive to outcome operationalization
Approval as outcome variable	142	No sufficient configuration satisfying all prespecified criteria identified	—	—	—	Sensitive to outcome operationalization
Comment threshold → 5	162	High Language Learning and Academic Adaptation added to the main configuration	0.930	0.285	0.813	More specific configurational variant
At most two videos per creator	125	Main configuration retained, with one additional configuration	0.901	0.428	0.800	Main structure retained
P05/P50/P95 calibration	142	High Language Learning and Academic Adaptation added to the main configuration	0.950	0.351	0.832	More specific configurational variant
Case-frequency threshold = 2	142	Main configuration unchanged	0.915	0.366	0.807	Configuration unchanged

## 5 Discussion, implications, and limitations

### 5.1 Discussion

#### 5.1.1 Audience responses to transnational experiential narratives present a multilevel structure of meaning

The findings show that audience responses to transnational experiential narratives by international students in China on the open digital platform of YouTube do not constitute a single attitudinal structure and should not be reduced to positive or negative evaluations of study experience in China. Instead, they involve different levels, including relational engagement, the practical use of lived experience, and broader meaning judgments. Existing research on cross-cultural digital communication has noted that audiences interpret and negotiate information provided by creators in relation to their own experiences and prior judgments (Li et al., 2025). This study further shows that such differences are reflected not only in the diversity of issues that concern audiences, but also in the ways the same type of transnational living experience may be placed within different practical needs and interpretive frames after entering the comment space.

More specifically, Relational and Affective Engagement indicates that audiences extend their attention from content to relational responses toward the creator as an individual. Educational Pathway Exploration, Study-Abroad Experiences in China, and Language Learning and Academic Adaptation show that international students‘ firsthand experiences can become experiential materials through which audiences understand study-abroad environments, reference personal choices, and recognize practical learning issues. At the same time, Study-Abroad Risk Appraisal and National-Frame Recontextualization indicate that some concrete experiences may be brought into judgments about study-abroad consequences, national differences, and broader social issues. The meaning of transnational experiential narratives after entering platforms is therefore not merely a continuation of the original content in comment sections, but may be reselected and reinterpreted according to audiences' own concerns.

This finding further enriches the cultural intermediary perspective on the communication processes of transnational individuals. Existing research has largely examined how actors with cross-cultural experience select, organize, and translate foreign living experiences from their own positions (Sullivan and Wang, 2024; Lee and Alhabash, 2025; Pan, 2026). The audience responses presented in this study suggest that the organization of experience by creators does not mean that the relevant meanings have been fixed. After the transnational living experiences provided by international students enter open platforms, they may also be used by audiences as resources for practical judgment, relational interaction, or social interpretation. The cultural intermediary role of international students should therefore not be understood only as the representation and translation of foreign experiences; it should also include consideration of how these experiences acquire further interpretation and use after entering audience spaces. The meaning of experience in transnational digital communication is thus better understood as an open process of meaning formation rather than as a result completed in advance and transmitted unidirectionally by creators.

#### 5.1.2 Theme retention and transition reflect changes in response focus during interaction

The thematic relationships between comments and replies further show that, after transnational experiential narratives enter audience spaces, their meanings do not remain within the initial responses formed by top-level comments. Instead, associated replies exhibit both thematic continuation and issue transition. Among the 5,211 valid pairs in which both the comment and reply had clear dominant themes, 52.7% of replies retained the original theme and 47.3% transitioned to another theme. This indicates that the discussion focus formed by top-level comments has a certain continuity, while a substantial proportion of responses also shift toward new issue directions. The degree of continuation varies across themes: Relational and Affective Engagement has the highest retention rate, National-Frame Recontextualization and Educational Pathway Exploration also show relatively strong continuity, whereas Study-Abroad Experiences in China and Language Learning and Academic Adaptation are more likely to transition toward other themes in subsequent replies.

More importantly, theme transitions show relatively concentrated directions. Study-Abroad Experiences in China often transitions toward Relational and Affective Engagement and Educational Pathway Exploration, indicating that discussions formed around concrete living experiences are likely in subsequent responses to connect further to the creator and to practical educational needs. Educational Pathway Exploration also frequently transitions toward Relational and Affective Engagement and Study-Abroad Experiences in China, showing that instrumental information needs do not always remain at the level of application or educational planning, but may re-enter personal relationships and concrete study-abroad experience. Language Learning and Academic Adaptation more often transitions toward Relational and Affective Engagement and Educational Pathway Exploration. Meanwhile, National-Frame Recontextualization, while retaining its own direction of discussion, also frequently transitions toward Relational and Affective Engagement and Study-Abroad Experiences in China. Discussion therefore does not move only in a single direction from concrete experience to macro-level issues; responses that have entered national-level interpretation may also return to individuals and everyday experience.

The overall net changes further demonstrate this interactional direction. Relational and Affective Engagement and Educational Pathway Exploration show net increases at the reply stage, whereas Study-Abroad Experiences in China and Language Learning and Academic Adaptation show net decreases. Concrete experiences of living and studying therefore more often function as starting points for subsequent discussion, while relational responses and educational information seeking show clearer convergence in replies. Research on dialogicality and recontextualization emphasizes that the meaning of responsive discourse depends on preceding expressions and specific interactional contexts, and that existing discourse may be reselected, reframed, and reorganized after entering new response relationships (Perrino, 2017; Gruber et al., 2023; Weizman, 2023; Jakob et al., 2024). The present findings locate this change more specifically in the thematic correspondence between top-level comments and associated replies. It should be emphasized, however, that the theme transitions identified here reflect changes in response focus within comment–reply relationships. They are not equivalent to the full process of discursive recontextualization and cannot be used to infer the continuous evolution of multiround interaction in the same discussion thread.

#### 5.1.3 Joint structures of multiple audience responses and high composite communication performance

Audience responses and platform communication performance do not show a relationship in which any one type of theme independently corresponds to higher composite communication performance. Necessity analysis shows that no single condition reaches the necessity threshold, regardless of whether the theme is in a high- or low-salience state. Further configurational analysis indicates that higher composite communication performance is associated with a specific joint structure of multiple audience response themes rather than with the isolated prominence of any one theme. The main analysis ultimately identifies one intermediate-solution configuration: high Relational and Affective Engagement, low Educational Pathway Exploration, low Study-Abroad Experiences in China, low Study-Abroad Risk Appraisal, and high National-Frame Recontextualization co-occur, while Language Learning and Academic Adaptation does not constitute a limiting condition in this configuration. Higher composite communication performance therefore does not correspond simply to the idea that more of a particular response type is better, but to a specific combinational relationship among different response themes.

A further comparison between the intermediate solution and the two equally parsimonious solutions clarifies the different positions of conditions within this combinational structure. Low Study-Abroad Experiences in China and high National-Frame Recontextualization are retained in both parsimonious solutions and are the most stable conditional features across different minimization schemes. This means that, in the present sample, higher composite communication performance more often appears in comment spaces where concrete discussion of study-abroad life is relatively non-dominant and where audience responses further enter broader national and transnational interpretive frames. Relational and Affective Engagement and Study-Abroad Risk Appraisal are each retained in only one parsimonious solution, so their core status has a degree of combinational dependence. The low salience of Educational Pathway Exploration appears only in the intermediate solution and is better understood as a contextual limit within a specific experiential configuration. The configurational results therefore do not show that several themes have fixed and universal effects, but rather reveal interdependence among different response conditions within specific combinations.

This result also structurally resonates with the preceding analysis of theme changes across comments and replies. Relational and Affective Engagement not only shows the highest theme-retention rate in the interaction analysis, but also forms a clear net increase at the reply stage and appears in a high-salience state in the main configuration. National-Frame Recontextualization changes relatively little overall between comments and replies, yet consistently appears with high salience in the main configuration and in both parsimonious solutions. Together, these findings suggest that higher composite communication performance often accompanies stronger relational responses and the involvement of broader meaning frames. This structural resonance does not mean, however, that relational engagement or national-frame discussion directly produces higher communication performance. Sensitivity analysis further shows that the main configuration has a degree of structural stability across changes in sample thresholds, creator repetition, calibration method, and frequency setting, but no configuration satisfying all sufficiency criteria is formed when Reach and Approval are used separately as outcome variables. The relationship identified in this study therefore mainly corresponds to composite communication performance jointly constituted by communication reach and audience approval, and cannot be further generalized to imply that a particular thematic combination necessarily corresponds to high reach or high approval.

The communicative associations of transnational experiential narratives are therefore better understood through the combinational structure of audience responses rather than through single-theme effects. The value of a configurational perspective here does not lie in identifying a fixed high-performance model, but in revealing how different response conditions co-occur in specific contexts and form set-theoretic associations with higher composite communication performance. Accordingly, the study presents configurational relationships between audience response themes and platform communication performance, and it cannot be used to infer that increasing a particular type of response will cause communication effects to improve.

### 5.2 Theoretical and practical implications

The principal theoretical implication of this study lies in extending the perspective of transnational digital communication from content production to the audience processes that unfold after content enters platforms. Existing research has largely examined how transnational creators select, organize, and represent foreign experiences from their own positions. This study further focuses on how those experiences are understood and used by audiences after public dissemination and how they take different response directions in subsequent interaction. Cultural intermediation consequently involves not only creators' organization and translation of transnational experiences but also the subsequent meaning processes formed after those experiences enter open platforms. By incorporating theme retention and transition between top-level comments and associated replies, the study moves audience research beyond static identification of what is discussed toward how existing responses are continued or how their focus changes in subsequent replies, thereby providing a more concrete empirical basis for understanding meaning negotiation in platform interaction. Furthermore, audience responses at the video level do not take the form of a one-to-one correspondence between a single theme and a communication outcome, but form configurational associations with higher composite communication performance through specific combinations of conditions. This extends audience analysis in transnational digital communication from individual textual content to three interconnected levels: theme formation, interactional change, and response configuration. It also addresses insufficient attention in existing research to comment-interaction relationships and combinations of multiple responses.

In practical terms, the findings suggest that transnational digital communication should pay closer attention to personal experiences grounded in concrete everyday life and to the actual responses those experiences receive after entering audience spaces. Transnational individuals should not be treated simply as instruments for transmitting predetermined national images or unified positions. The experiences formed through international students' long-term study and life in China can provide different audiences with concrete references for understanding study-abroad environments, educational choices, and social realities. Once such experiences enter open platforms, however, they may further generate relational engagement, practical information use, risk judgments, and broader social and national-level interpretations. Communication practice should therefore fully consider the diverse understandings that audiences may form from the same experiential content. Comment sections should not be regarded merely as auxiliary indicators of interaction volume, but as part of the communication process itself. By answering questions, supplementing background information, and maintaining exchanges, creators can provide more experiential information for subsequent discussion, but audience interpretations remain shaped by their own needs, experiences, and prior understandings. Communication evaluation should likewise not judge whether content is effective on the basis of the prominence of a single response theme. Beyond platform indicators such as views and likes, it should also attend to what kinds of responses audiences form, how these responses are retained or shifted between comments and associated replies, and how different response themes jointly constitute a specific discussion space. This approach allows a more complete understanding of actual audience processes and communication performance in transnational digital communication on open platforms.

### 5.3 Limitations and future research

This study still has several areas for further development. The sample consists mainly of videos by international students in China on YouTube and was formed through purposive retrieval and screening, with videos having richer comment interaction enjoying relatively higher visibility. The findings are therefore better suited to understanding the audience response structure within this specific platform sample, and should be tested with additional materials in other platforms and communication contexts. In addition, the formal analysis is based mainly on high-confidence English texts. Non-English and code-switching interactions are not fully represented, although such multilingual expressions may themselves contain different cross-cultural experiences and interactional meanings. Topic identification and comment–reply analysis also have methodological boundaries. Model classification and a unified thematic framework help form a comparable analytical basis, but new themes that may emerge at the reply stage and more complete multiround interactional processes require further examination. Video-level configurational analysis also varies to some extent across different sample thresholds, calibration methods, and operationalizations of communication performance, so the results are best understood within the sample and composite communication performance framework set by this study. Moreover, because the study is based on platform data observed at a unified time point, it mainly reveals associations between audience response structures and communication performance. It is difficult to further determine temporal ordering or possible mechanisms, and video content, creator characteristics, and platform recommendation mechanisms may also be involved. Future research can expand to different platforms, language groups, and communication contexts, and combine complete reply chains, multilingual text analysis, and multi-time-point communication data to observe more continuously the formation and change of audience responses after transnational experiential narratives enter platforms and their dynamic relationships with communication performance.

## 6 Conclusion

After transnational experiential narratives by international students in China enter YouTube, their communicative meanings present different directions of understanding and discussion in audience responses. Audiences may understand such content as a medium for establishing relationships with creators, or they may transform it into a reference for understanding study-abroad environments, planning educational choices, judging risks, or interpreting broader social and national issues. Transnational experiential narratives therefore do not carry a single meaning fixed in advance, but constitute experiential resources that different audiences can reuse according to their own concerns. Theme continuation and a large number of theme transitions both appear between comments and associated replies, further indicating that audience responses on platforms are not isolated collections of opinions, but continuously adjust their discussion focus through interaction. Particularly noteworthy is that concrete experiences of living and studying more often serve as starting points for subsequent discussion, while relational responses and educational information needs are more likely to converge during the reply process. This shows that, after transnational experiences enter platforms, their meanings often move from what is narrated toward for whom these experiences are useful, how they are understood, and how they continue to be discussed.

Furthermore, high communication performance does not simply depend on the prominence of one type of response, but is more likely related to how different levels of meaning are jointly organized in the comment space. Overall, the findings show that concrete study-abroad life experience is not the endpoint of communicative association. Higher composite communication performance more often appears in comment spaces where concrete discussion of study-abroad life is relatively non-dominant and where national and transnational interpretive frames are more prominent. Relational and Affective Engagement also appears as high salience in the intermediate solution, although its core status has a degree of solution dependence. The communicative value of transnational experiential narratives therefore lies not only in providing concrete firsthand personal experience, but also in whether such experiences can be further transformed by audiences into relational connection, practical judgment, and broader meaning interpretation. Understanding transnational digital communication consequently cannot stop at how creators represent foreign experiences. It must also attend to how experiences are taken up, transformed, and organized into new discussion structures after entering platforms. Transnational experiential narratives are thus better understood as an open communication process jointly constituted by creator expression, audience interpretation, and subsequent interaction. Their communicative meanings are not the result of one-way output, but are gradually generated through sustained audience participation.

## Data Availability

The raw data supporting the conclusions of this article will be made available by the authors, without undue reservation.
